# Community-based assessment of human rights in a complex humanitarian emergency: the Emergency Assistance Teams-Burma and Cyclone Nargis

**DOI:** 10.1186/1752-1505-4-8

**Published:** 2010-04-19

**Authors:** Voravit Suwanvanichkij, Noriyuki Murakami, Catherine I Lee, Jen Leigh, Andrea L Wirtz, Brock Daniels, Mahn Mahn, Cynthia Maung, Chris Beyrer

**Affiliations:** 1 Johns Hopkins Bloomberg School of Public Health Center for Public Health and Human Rights 615 N Wolfe Street Baltimore, MD 21205, USA; 2 Emergency Assistance Team (Burma) PO Box 57 Mae Sot, Tak 63110 Thailand

## Abstract

**Introduction:**

Cyclone Nargis hit Burma on May 2, 2008, killing over 138,000 and affecting at least 2.4 million people. The Burmese military junta, the State Peace and Development Council (SPDC), initially blocked international aid to storm victims, forcing community-based organizations such as the Emergency Assistance Teams-Burma (EAT) to fill the void, helping with cyclone relief and long-term reconstruction. Recognizing the need for independent monitoring of the human rights situation in cyclone-affected areas, particularly given censorship over storm relief coverage, EAT initiated such documentation efforts.

**Methods:**

A human rights investigation was conducted to document selected human rights abuses that had initially been reported to volunteers providing relief services in cyclone affected areas. Using participatory research methods and qualitative, semi-structured interviews, EAT volunteers collected 103 testimonies from August 2008 to June 2009; 42 from relief workers and 61 from storm survivors.

**Results:**

One year after the storm, basic necessities such as food, potable water, and shelter remained insufficient for many, a situation exacerbated by lack of support to help rebuild livelihoods and worsening household debt. This precluded many survivors from being able to access healthcare services, which were inadequate even before Cyclone Nargis. Aid efforts continued to be met with government restrictions and harassment, and relief workers continued to face threats and fear of arrest. Abuses, including land confiscation and misappropriation of aid, were reported during reconstruction, and tight government control over communication and information exchange continued.

**Conclusions:**

Basic needs of many cyclone survivors in the Irrawaddy Delta remained unmet over a year following Cyclone Nargis. Official impediments to delivery of aid to storm survivors continued, including human rights abrogations experienced by civilians during reconstruction efforts. Such issues remain unaddressed in official assessments conducted in partnership with the SPDC. Private, community-based relief organizations like EAT are well positioned and able to independently assess human rights conditions in response to complex humanitarian emergencies such as Cyclone Nargis; efforts of this nature must be encouraged, particularly in settings where human rights abuses have been documented and censorship is widespread.

## Introduction

**Figure 1:**
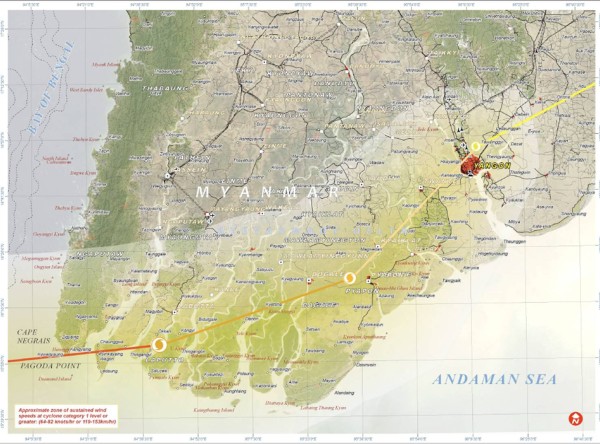
**May 4th 2008, Cyclone Nargis path retraced with view of areas affected by Cyclone Nargis in the Irrawaddy Delta, Burma (UNOSAT)**[62].

Cyclone Nargis hit Burma's Irrawaddy Delta on May 2, 2008 (Figure 1), killing over 138,000 and directly affecting at least 2.4 million more[1,2]. A storm of this magnitude poses challenges to any government; however, Cyclone Nargis hit Burma (also known as Myanmar), a country impoverished under decades of military rule and with decimated health and education sectors, and collectively rendered this ill-prepared country unable to recover after a crisis of this scale [3-5]. Following the cyclone, a humanitarian crisis ensued, one which arguably became a complex humanitarian emergency (CHE), defined as "a humanitarian crisis in a country, region, or society where there is total or considerable breakdown of authority resulting from internal or external conflict and which requires an international response that goes beyond the mandate or capacity of any single and/or ongoing UN country program" [6-8]. The Burmese regime, the State Peace and Development Council (SPDC), initially refused international aid; refused to lift visa restrictions for humanitarian workers; and used state resources, including troops, to support a scheduled referendum on a military-backed constitution [9-13].

As international pressure mounted, the regime began to allow some access by international aid agencies, particularly following an unprecedented visit to Burma by UN Secretary General Ban Ki-moon on May 22-23^rd^[14]. Concurrently, the Tripartite Core Group (TCG), composed of the UN, ASEAN, and the SPDC, was formed and became the lead entity for the Nargis Response[15].

### The Emergency Assistance Teams Burma

While most international efforts to aid storm survivors were stalled, local community-based organizations and individuals were often the first-responders[16]. Within three days of the storm's landfall, the Emergency Assistance Teams-Burma (EAT), a community-based network of organizations and individuals, was formed and began providing relief to cyclone survivors. EAT volunteers, mostly cyclone survivors themselves and unaffiliated with the regime, received aid donated by communities living along the Thai-Burma border (in Burma or Thailand), as well as through international organizations that sent aid through Thailand. Members of the relief teams, eventually totaling 44 teams of several volunteers per team, also received training in Thailand on emergency response, food and water distribution, and basic first aid provision, and with donated supplies were able to quickly provide assistance to some of the hardest-hit communities in the Irrawaddy, Rangoon, and Pegu Divisions. Within the first three months, EAT delivered aid to over 180,000 survivors living in 87 villages of 17 townships, providing essential assistance such as clean water and food, clothing, and shelter; assisting in proper disposal of corpses; facilitating family reunification; and providing emergency healthcare. In the second phase of the EAT response, from August 1, 2008, to January 31, 2009, the teams continued to provide water and food aid, but also focused on rehabilitation efforts, including the rebuilding of homes and aiding in the re-establishment of livelihoods, education, and health infrastructure.

Widespread violations of fundamental freedoms and human rights perpetrated by the SPDC have been well-documented [17-19]. Within weeks of the storm, independent organizations [20-22] and the media began to report human rights abuses in cyclone-affected areas, including forced relocation of survivors, restrictions on humanitarian aid, and confiscation and diversion of aid[12,23-26]. Official assessments, including those conducted with the SPDC, generally did not address these concerns[16,27]. This reality convinced EAT and its partners that an independent assessment of the human rights situation in the affected areas was needed; information vital both for informing comprehensive program planning and policy but also for community empowerment and freedom to participate in reconstruction efforts.

In February 2009, EAT and its partners released a report "After the Storm: Voices from the Delta," documenting human rights violations in the wake of Cyclone Nargis[28]. The report generated significant controversy, particularly with respect to the nature of the assessment itself. EAT had performed a human rights assessment using qualitative human rights methods, that included in-depth interviews with relief workers and cyclone survivors. However, the report was widely viewed as an assessment of the overall humanitarian response, for which the methods used would have been inappropriate. EAT members were misconstrued as being from the Thai-Burma border areas or otherwise not recruited, as they had been, from affected Delta communities. And the report was represented as a call to limit humanitarian assistance, although no such call was made in the report or its recommendations[28].

### The Later Phases of the Response

Human rights abuses continued to be reported during the latter phases of the Nargis response. Independent relief workers continued to be arrested and imprisoned, including an additional five independent donors and ten relief workers, detained in September-October 2009 [29-31]. Meanwhile, the regime's contribution to relief and rebuilding efforts continued to remain limited. In September, 2009, the UN Human Settlements Programme (UN-HABITAT) estimated that some 450,000 people in the Delta were still in dire need of shelter 18 months after the storm; the junta had constructed some 10,000 houses[32], international donors built some 25,000 houses, while the Burmese people themselves had built 209,000[32,33]. Towards the end of 2009, while the SPDC spent over $570 million on advanced fighter jets from Russia [34,35], the TCG appealed for $103 million for priority reconstruction initiatives, of which only $88 million was pledged by the international community [36-38].

Starting in May, 2009, EAT conducted an additional round of interviews with relief workers and cyclone survivors to assess the human rights situation during the later phases of the relief effort, one year after the storm. The findings presented here include personal accounts from interviews conducted during the earlier phases of the response and from later rounds of data collection, accounts not included in "After the Storm."

## Methods

A collaborative group was formed to conduct a community-based human rights assessment, which included EAT, the Mae Tao Clinic, local human rights organizations, and the Johns Hopkins Center for Public Health and Human Rights, which contributed technical support for training community investigators, developing survey instruments, training in interview methods and conduct of human subjects research, and provided support for data analysis.

Qualitative research methods were used, as they allow for detailed comparison of the experiences of survivors and relief workers and, given the security situation and the arrests of several prominent citizens engaged in independent relief work [39-41], such methods were also the only feasible approach for conducting human rights investigations with minimal risk to participants and interviewers. One-on-one interviews were therefore employed to collect in-depth data from survivors and relief workers. Three rounds of data collection were undertaken, the first from June to September 2008, then in October to November 2008, and May to June 2009, to gather additional information on the later phases of the response. Using purposive sampling, 103 in-depth interviews, 42 with relief workers and 61 with cyclone survivors, were conducted in Burma's Irrawaddy and Rangoon Divisions as well as along the border with Thailand. In all, 87 communities in 17 townships were represented by at least one interview.

Interviewers were recruited from members of EAT's community networks that were providing emergency relief inside the Irrawaddy Delta after the cyclone. Interviewers were recruited on a voluntary basis and were chosen for their knowledge of and access to the communities, particularly those hardest hit.

The interview format utilized participatory research methods and in-depth qualitative interviews focusing on selected rights abuses which were identified during preliminary formative research[42,43]. The selected relief workers were trained in Thailand to conduct interviews. Trainings focused on case finding, interviewee confidentiality and security, informed consent, screening candidates for interviews, open-ended qualitative interviewing skills, accurate and secure data gathering techniques, contact-based sampling methods, and human rights principles. Refresher training sessions occurred regularly, as relief workers returned to the Thai border for re-supply of aid materials. Local human rights organizations, including the Karen Human Rights Group, provided assistance during the trainings.

The qualitative interview format for the relief worker interviews was developed for the assessment based on initial key informant interviews with relief workers during the first month after the cyclone. Interview domains were developed along with specific probes through an iterative process that incorporated input from study team members and other leaders from organizations operating in this environment. The guide was then piloted with several local team members and refined for clarity, ease of use, and brevity. The interview guides and consent scripts were translated into the three most commonly used languages in survey sites: Burmese, Skaw- and Pwo-Karen. Domains covered internal displacement; discrimination in provision of relief; community responses; and personal security and logistical concerns. Later, domains were added to explore topics of child labor, security and other concerns related to women and debt, as these issues emerged during recovery efforts.

A similar process was used to generate a qualitative instrument for interviews with Nargis survivors. The EAT team decided that only adults (18 years or older) would be interviewed, as it was difficult to assess the agency and protection for children in Burma. A semi-structured instrument that utilized a flexible set of open-ended probes was developed to elicit in-depth information about human rights concerns. Domains for survivor interviews included questions about the survivor and his/her family before the cyclone; experience during the cyclone; knowledge or warning of the impending cyclone; the situation and events experienced by the survivor and his/her family immediately post-landfall until the day of the interview; negative experiences; and plans for the future. During the second phase of the assessment, much of the survivor interviews in the affected areas focused on probing further into personal experiences of human rights abrogation and protection. Interviewers were trained to utilize pre-designed screening questions to aid in the identification of candidates with detailed primary information regarding any one of the human rights abuses. Once potential candidates were identified and oral informed consent had been obtained, the confidential and anonymous interview was conducted in a secure location and the set of open-ended probes for the relevant domain(s) were then employed by the interviewer to continue gathering detailed and sensitive information. During the interview process, detailed questions or probes were used to elicit further details of experiences, a process that serves both to gather the most information possible as well as to ensure internal consistence and to differentiate personal experiences from hearsay.

Because of the risk associated with collecting this information in Burma, interviewers took numerous precautionary measures to ensure the protection of both the participants and themselves. No personal identifying information was collected and, prior to participation, oral scripts to obtain verbal consent were used- as opposed to signed consent forms- to prevent unintentional revelation to outsiders of an individual's participation in the study. Interviews were recorded using a portable digital recording device after obtaining the participant's explicit permission regarding its use and after providing basic instructions to omit details that might reveal the identities of the survivors or their communities during the interview. Interviews were identified by the date and location of the interview, logged in a simple string of numeric codes, a method that also ensured protection of interviewer and interviewee.

Interview data was sent to a central location where they were translated into English, transcribed, and re-checked for accuracy by bilingual EAT staff. The English language transcripts were then analyzed by the JHU team using qualitative analytic approaches. The data from the interviews were analyzed based on relevant human rights themes, chronology of the event(s), location, demographics of affected communities, demographic information of interviewee, and details of government, military and NGO involvement. Data were then analyzed for widespread patterns and differences using a modified qualitative matrix. At the time of final reporting, any additional information that was believed to potentially place the interviewer or interviewee at risk of identification was removed to further ensure protection.

Ethical approval for this study was granted by the Institutional Review Board (IRB) of the Johns Hopkins Bloomberg School of Public Health, and by the Burma Medical Association's Ethical Committee.

## Results

In total, EAT teams conducted 103 in-depth qualitative interviews; 42 were with relief workers from cyclone-affected areas, 61 were with Nargis survivors. Of these, nine relief worker interviews and four survivor interviews were conducted in Thailand during the latest round of data collection, in May-June 2009. Names of participants and villages, as well as other identifiers that were revealed during interviews have been removed for the security of participants and members of their communities. Findings from these interviews are summarized in Table 1.

**Table 1:** Summary of findings from interviews with cyclone survivors and relief workers:

Domain	Concern
**Basic necessities**	- Survivors lacked clean water and food - Distance and cost of obtaining and transporting were challenges that had to be met, often at the sacrifice of meeting other needs - Shelters and new homes were inadequate; many residents were still homeless or in temporary housing - Concerned with the stability and protection provided by the new structures

**Health**	- Diarrhea and illnesses related to water shortages persisted - Noted psychological disorders associated with traumatic event of the storm and loss - Basic health services remained insufficient (a reality even before the cyclone)

**Government interference (direct and indirect)**	- Check-points were in place along routes into the Delta during the early phases of response - "Fees" were charged to access disaster areas - Travel restrictions occurred - Aid workers were extensively monitored - Relief workers were required to give aid directly to the authorities - Relief and reconstruction materials were misappropriated

**Security Concerns**	- Intimidation, abuse, and fear of arrest of relief workers - Security concerns further obstructed the delivery of aid to cyclone victims

**Information**	- Challenges and security concerns were associated with collecting information - Needs assessments and coordination of relief activities particularly were hampered by inability to independently collect data and communicate - Information released through the state-controlled media outlets minimized the extent of the disaster and needs of the victims

### Needs for Food, Water, and Shelter following the cyclone

A year after the Cyclone, respondents reported that basic necessities remained unavailable for many[44].

The people in my area still need boats and fishing nets. We also need some small shops in order to sell noodles and basic supplies.....We lost everything in the whole village - our house, our belongings, our buffalos, and cows... So now we need these things to rebuild. **Survivor, Female, from Labutta Township, Irrawaddy Division (May 3, 2009)**

Food supplies, particularly agricultural outputs, were insufficient in quantity and quality due to the immediate destruction of land and crops by the cyclone and exacerbated by the loss of farming equipment, increasing debt, and interruption of labor, as survivors were forced to prioritize other needs, such as acquiring water or rebuilding shelters, over farming.

One of the small villages that I visited, I saw that they didn't have any assistance from anyone so they still needed houses. And for the farmers, they also need farming tools and other things like paddy seeds and petrol. The people also need food because they cannot survive on their own now and some people are still without a job. They have a lot of difficulty for getting food. The reason they don't have houses yet is that there is not an organization working there or a donor. Also, they are busy searching for food so they don't have time to work on building houses and also there is no extra money for building the houses.** Relief worker, Female, from Labutta Township, Irrawaddy Division (May 8, 2009)**

...they [cyclone survivors] are daily workers, finding enough food is a big problem. They don't have money to buy food so sometimes they have to borrow from the other houses. The main issue is that they don't have money to buy the food that is available in the village. **Relief Worker, Female, from Labutta Township, Irrawaddy Division (May 8, 2009)**

Access to potable water remained problematic; a consequence of wells and ponds still contaminated by saltwater as well as the end of the monsoon rains.

For water, we have to go to a village that is one day walking away. We have to stay in that village one night and then come back. There is a big problem for getting water and food in our village. We have to search for a long time for some plants that we can sell and get other things, other food....Even now, even today we still have to get water this way. **Survivor, Female, from Labutta Township, Irrawaddy Division (May 3, 2009)**

For the water, they tried to repair a pump but 1 week after they fixed it, they found out that the water was not good so this affected their health. Also, there is a water purifications machine, but this is not working well either...Now we do a water project and the authorities and village leaders say that the emergency period is over so they said that we don't need to support these livelihoods projects. **Relief Worker, Female, working in Rangoon, Dedaye, Labutta and Mawlamyinegyun (May 5, 2009)**

The emergency need right now is water. They do not have water in the village so they must [travel] by boat 6 hours one way in order to get water. We also have to purchase the water from Labutta. For these people, they are the ones who are poor already and so they cannot afford it and it is really difficult for them. They must spend money on the water so it is difficult for them to purchase enough food. **Relief Worker, Male, working in Labutta Township, Irrawaddy Division (May 5, 2009)**

Shelters remained inadequate, and many residents were still homeless or living in temporary housing. Among those who had their houses rebuilt or were provided with new ones, several voiced concerns for the stability and protection provided by the new structures.

In my village about half of the people have been able to rebuild their houses, but the other half have not been able to. The government said they would build the houses for the village, but what they distributed was not enough. Some people were able to build, but not everyone. For the people who did not receive support they had to make a shelter out of bamboo and tarpaulin, but it is not safe enough to protect them. Even the people who received housing, though, the houses are not of good quality. When it rains, the roof leaks and also the walls cannot protect against the rain. So, both the people who have houses and temporary shelters are wet from the rain. **Relief Worker, Female, Working in Labutta (May 5, 2009)**

They [cyclone survivors] have not been able to build their houses because they do not have enough money to rebuild. For the food, they don't have enough money to buy enough food, because they are daily workers. For the people who have not build a new house yet, some build their houses in another person's garden. Some other people built shelters out of tarpaulin. But, these houses are too hot and when it rains it does not protect them from the rain. **Survivor, Female, from Kungyangon Township in Rangoon Division (May 4, 2009)**

A year after the storm, issues such as diarrhea and illnesses related to water shortages, as well as psychological disorders, were noted[45]. Basic health services remained insufficient for many survivors, a reality even before the cyclone. Local relief workers and clergy often received training and provided basic health services and, in some cases, medicine when this was possible.

But we see a lot of children with diarrhea. The villagers cannot do anything when the child has diarrhea. They would need to go all the way to Labutta because there is nothing to treat them within our village. **Survivor, Female, from Labutta Township, Irrawaddy Division (May 3, 2009)**

For medicine, even though we support them, we don't have enough medicine to meet their needs. Also, there are no health workers, nurses or midwives in these villages so the people have to go to another village to get services. Even though we cannot provide a doctor, we work with a doctor to get the medicine and learn how to use it and then provide it to them. **Relief Worker, Female, Working in Rangoon, Dedaye, Labutta and Mawlamyinegyun (May 5, 2009)**

For the women, the pregnant women, we don't have enough medicine to take care of them very effectively. We can only talk with them, but we cannot provide treatment and this makes it difficult for us as health workers and also for the women. To go to the township, it is too far to go. Most of the people have to stay in the village. By boat it is 7 hours. In my village we do not have a clinic, but there are the three of us health workers...We have a public health team. These people provide training and information to the people about boiling their water and what to do when they have diarrhea. They also show them how to use ORS properly. These people just organized by themselves to do this, they are not an organization...For us, we are from the rural areas and travel is very difficult so I hope someone will help us by providing donations for medicine and assisting us with rehabilitation. The medicine is the most important because it is very difficult to travel to the township, especially during the rainy season. We have to cross the sea to get to the township and it is difficult and dangerous. **Relief Worker, Female, Working in Labutta Township, Irrawaddy Division (May 5, 2009)**

Some children in the area also have a lot of coughing and I think it is TB. They don't have any place to go and get tested to see if it's TB, though. These places are so far away, too far away. It takes one day to walk there, the whole day. Some of the children cough for 1-2 months every day and then the parents make the trip to the town for testing. The situation now and before Nargis is different because now they cannot go to the township. Even if they have a clinic in the village, there are not enough health workers and doctors. Even if you want to go to that clinic, you have to pay 5,000 kyat [approximately $4 USD] for one visit, but this does not include the medicine costs... But, it is also the case that before Nargis there were no health workers or doctors...In our village, there are 2 clinics. One from the government, and one private. If you want to go there, you have to pay. There are no free services. Most people are not able to go. Most people cannot afford to pay. If it is not serious, they do not go to the clinics, they just buy some medicine from the pharmacy or small shop, but this is not western medicine, it is only traditional medicine.The situation is the same now as it was before [Cyclone Nargis]. **Survivor, male, from Kungyangon Township, Rangoon Division (May 3, 2009)**

The health situation does not seem to be so bad. It is the same situation as before the cyclone. There is no clinic in our village so people have to travel about 20 minutes by trishaw. If there is a big problem then people go there, but if it's not to major then we get treatment from one of the monks. **Relief Worker, Female, Working in Rangoon Division (May 4, 2009)**

### Government Interference in Relief Efforts

Although delivery of aid was hampered by difficulties in physical access and travel, government interference, direct and indirect, was also frequently reported. These included travel restrictions, check-points along routes into the Delta, "fees" to access disaster areas, extensive monitoring of aid workers, and the demand for aid to be given directly to the authorities. Many of these challenges persisted into 2009.

Around February or March 2009 the authorities asked us where we got permission to work in these areas and who supported us. Even though we are a social group, we had to lie and say we were from [name deleted] because the [name deleted] gave us support to do anti-trafficking work. They support funds for the livelihoods project and the anti-trafficking training work. But, the authorities told us that the emergency period is over and most of the NGO already went back so when we work there the authorities ask us many questions.... Even though they don't want us to do this work and even though the support has stopped from [name deleted] we still do this work and when they ask us about our work we lie and say we are with [name deleted]. Now we only receive support from our friends outside of Burma We have to do this for our safety. We have to do this because if the authorities found out exactly what we are doing it would depend on the authorities what happens to us... For me, I am not afraid of being arrested, but I want to continue to do this work and complete my work. If they know about our project and stop us then we cannot do this work. I have a good relationship with the villagers and for their long-term plan we have already arranged everything and we need to work together. When we go there we understand each other and work together. I have heard of people being arrested for doing similar work, but I don't know any of the exact details. **Relief Worker, Female, Working in Yangon, Dedaye, Labutta and Mawlamyinegyun. (May 5, 2009)**

Now INGO work is still happening, but it is a very restricted condition. When we first started working, there were no INGOs there. They came about 1.5 months after the cyclone. But now, their movement is restricted, especially if they don't have an MOU [Memorandum of Understanding, with the Burmese government], so they have to draw back. All 10 villages that we work in have INGOs present, but they come less often. We can move quickly and freely, but the security situation for us is worse than for those with an MOU. They have to wait for permission to do things, but we just go and do them, but we have to be careful. We coordinate and talk with INGOs, though. There are coordination meetings that take place. For things like human rights violation issues, though, we talk about this outside of the meeting because we have to be careful of security and who is listening. The INGOs seem to listen to the problems, but they don't take any action. We have tried for registration, but because we are a [ethnicity deleted] group and a women's group, we think that is why we have been denied. INGOs are allowed to openly coordinate with each other and with the government, but we have no direct communication with the government and we are restricted in how we can work with the INGOs because we have to keep quiet. **Relief Worker, Female, working in Dedaye, Moulmeingyun, and Labutta (May 14, 2009)**

### Confiscation of Relief Supplies

Concerns about misappropriation of relief and reconstruction materials continued in 2009:

For these people, even though we heard that the NGOs will donate houses, they cannot go directly to the village, they have to go through the government and the government also does the contract to have the houses built so the house that were built were not enough for everyone in the village. Even the NGO made it to our village; they only provided a small amount of food and clothing support, but what the village really needs are support for their livelihood. **Relief Worker, Male, working in Labutta Township, Irrawaddy Division (May 5, 2009)**

Last year there were NGOs working in this area, but they left this year in March... Around April 15th or 16th, I didn't see any organizations at that time. This village still needs support from the NGO, but the NGO said they cannot do this work freely and directly to the beneficiary and they have to work through the government so they don't want to come again. People from the authorities are very involved in the contract with the NGOs [names of 5 INGOs deleted]. In the places I work, they stopped food support in February 2009. For the villagers, even though know that the NGOs support them, but they don't get it or they get only small things. The house that I have, it was cheaper than the contract that the government had and built and my house is still in good condition, while the house provided by the government is already damaged. **Relief Worker, Male, working in Labutta Township, Irrawaddy Division (May 5, 2009)**

The other example was when some people donated clothes, the good quality ones were taken by the village leader to give to his family and friends. The ones of poor quality were given to the poor people. Also, when diesel was given to the village, it was supposed to be given to the whole village, but actually he only gave it to the farmer and then sold the remaining to other villages to make money. the village leaders are not chosen by the people in this area, they are appointed by the government and are part of the government. In almost all of the villages I have visited, I see corruption on the part of the village leader. **Relief Worker, Female, working in Labutta Township, Irrawaddy Division (May 8, 2009)**

I saw that all of the assistance had to go through the government, it cannot go directly. Because of this, when it actually reaches the community, there is some missing. One example is for the housing, the supplies were donated and then the government took the supplies and hired a company to build the houses. The authorities told the donor it will cost 2,000,000 kyat [approximately $1667 USD], but actually it costs only 500,000 [approximately $417 USD]. The government charges the donor for 2,000,000 but only spends 500,000 on the house and keeps the rest... **Relief worker, Male, Working in Labutta, Bogale, and Mawlamyinegyun Townships, Irrawaddy Division. (May 8, 2009)**

### Arrest of Relief Workers and Security Concerns

Obstruction in the delivery of aid to cyclone victims also occurred as a result of intimidation, abuse, and arrest of relief workers, especially private volunteers. Several interviews revealed that relief workers often had to provide some form of bribery in order to work.

After one month, they came to the village, saw my supplies and started asking - they sent my information to Yangon [Rangoon] to investigate me. They were asking why there were so many supplies. They think it was anti-government. So I left; I don't like prison. **Relief Worker, Male, Physician, working in Pyapon Township, Irrawaddy Division. (August 20, 2008)**

Before I go, I always plan for my security and check to make sure everything is ok and then I go to do work. If I see some problem, authorities, I already have a plan of what I have to say or what I have to do. For this, one problem is if they check us. When we distribute assistance, we cannot let them see this assistance and we go quietly. They don't know I do relief work in this area. If you can explain very clearly, maybe it will be ok, but if I am caught I could be arrested. **Relief Worker, Male, working in Labutta Township, Irrawaddy Division (May 5, 2009)**

Only the village leader knew about this youth group and their support so there is no problem. The village leader does not talk about their work to the other authorities. He does not explain how the community works to support themselves. I think that if the authorities know about this, the situation could be worse. I think that the authorities don't like when people organize themselves or make some kind of group... The people in the village think that it is best if I get permission and maybe they won't know where I have studied and worked, and then they will give permission for me to do this work. My cousin advised me to give the authorities some money to make everything okay. When I go back to work, I have a plan to talk to the authorities. My cousin is one of the clerks from that area so she knows the situation very well and she offered to take me to the authorities. She said we will go together and give some presents to the authorities and then they will let me go to the village. **Relief Worker, Female, Working in Labutta Township, Irrawaddy Division (May 8, 2009)**

For my staff, they face a lot of problems in their work. It is difficult for them to travel and also they have problems with security. There are also problems with poor communication, threats to the group, and also long travel. On April 16 in [name deleted], a soldier from LIB [Light Infantry Battalion] 613 severely beat one of my relief worker staff. The soldier said that this was because he didn't have travel permission. But we have been working there for years and also since the cyclone and no one mentioned about needing travel permission for people from there. There was no announcement. He was severely beaten especially around his head and face. This town is a military check point. But before the cyclone it was not. Before the cyclone there was no military here. Now, they collect a tax of 500 kyat [approximately $0.40 USD] on every boat and 1000 kyat [approximately $0.80USD] on larger boats that go through this town. **Relief Worker, Female, Working in Dedaye, Moulmeingyun, and Labutta Townships, Irrawaddy Division (May 14, 2009)**

### Information

Difficulties in collecting and accessing reliable information, particularly that which was necessary for needs assessments and coordination of relief activities, was a major concern for relief workers. This was exacerbated by information released through the state-controlled media outlets, which frequently minimized or obscured the extent of the disaster or needs of the victims to create the impression that the government's relief efforts were meeting the needs of survivors.

For the foreigner groups the authorities prevent them from going to certain places and actually seeing the situation in the villages so they cannot actually get information from the people, only from the authorities. For us, we go as a church group and we are from that place so we can sit and talk with people... The [Burmese] representatives of the donors came to the village because if the donor is a foreigner then the authorities say there is some security problem and the foreigner cannot go. They send a representative again. The authorities allowed the representative to take photographs, but only from far away, no pictures up close and only from the front view of the house. And the representative was not allowed to talk with or ask questions of any of the villagers. The reason for the pictures of the front view is because they do the walls correctly in the front, but on the sides and the back, as well as the roof, they leave wide cracks in the walls. The floors also are not made well. Even me, because I am a little big, I am afraid to go into the houses because it is very thin and it might become broken. Now some houses are already damaged. **Relief Worker, Male, working in Labutta, Bogale, and Mawlamyinegyun Townships, Irrawaddy Division (May 8, 2009)**

The first time I went, the authorities knew we were going there and we didn't have any problems. But, the next time we went, we went a different way to get to the village and we had some problems. We had three teams and three boats. On that way, one of the teams met with soldiers from the army and were stopped and questioned. But the other 2 came back to the base office in time. When we all arrived back in the place, in [location removed], the police called all of us together to investigate. They brought us to the playground. They asked us where we were from and what we were doing, which organization we were with, where we were going. They accepted the relief aid, but they didn't want us to collect the lists of information on the population. They told us to stop collecting the information on population and the health situation... with the other authorities, we didn't meet any problems with them because the villagers gave us information on where the soldiers were so we could avoid them. We avoided them because if we met them they would surely ask us questions about our work. For me, I didn't have any problems with my security. For our youth group, we don't worry about any situation for us, but for our leaders, they worried about our safety and security so they decided to stop our work. They were afraid that the government authorities would arrest some of us or all of us in the group for providing these services or because we were documenting population information. **Relief Worker, Male, working in Labutta Township, Irrawaddy Division (May 7, 2009)**

In every village now the authorities have placed members of the "Swan Arr Shin" [Masters of Force] or "Kyant Phut" [Union Solidarity and Development Association, USDA; both are para-statal organizations implicated in violent attacks on regime opponents] group in each village. They try to make them look like regular people, but really they are people from the government, from the army. What they do is the authorities have these people control the village, listen to what is happening, try to stop any activities they don't like and they hope that it looks like regular villagers stopping each other, but really it is the soldiers. If someone comes to donate something, they tell the donor that because of security they will do it themselves, but really it is to take the donation and be able to control how it is used or how the money is used... Only the villagers know who these people, these informers, are but the donors don't know. They told the villagers not to tell the donors who they are. They said that if the villagers told the donors about them, they would "take action." They want everyone to think that they are from the community, that they are normal villagers. They said that if the villagers tell anything about the work of the "suan ahn chin" that group will stop all aid coming in and will arrest the people responsible. They also threaten the community before the donors come to see the donation of the houses. They told the villagers that if the donors ask anything, they have to say everything is good and perfect and if they said any of the weak points, they would take back the house and then arrest them because the army came with the donor. This practice of placing people from the army in plain clothes in the villages started just after the cyclone and still continues today. **Relief worker, Male, Working in Labutta, Bogale, and Mawlamyinegyun Townships, Irrawaddy Division (May 8, 2009)**

### Distribution of Aid

Reports of discrimination in the delivery of aid as well as misappropriation of aid was also often recounted by respondents; in particular, supplies were required to go through official channels for distribution, likely resulting in a significant amount of donations not reaching its intended recipients:

In one village, they had a project to build the houses and when the materials arrived, the village leader announced to the village that 1 person per family had to go and help carry the items. For some people, they didn't have time to go so they have to find someone and pay them to go in their place. This was a donation..., they had to carry it to the village leader's house. Then the company that came to build the houses they went to use these materials to build the specific houses, as I said, these were for the friends and family of the village leader, the supplies were not shared among the community. This was also in [name deleted] in Labutta. They had to carry these supplies 2-3 days and received no food or money for this work. **Relief worker, Female, Working in Labutta Township, Irrawaddy Division (May 8, 2009)**

Also, some people decided to leave because they had no place to live so they decided to go away. But, when they tried to come back, the village leader said they could not come back because, after Nargis, they had already left. In this situation, when they temporarily lived in another village, that village did not want to accept them. They told them to go back to their own village, but when they went back to the original village the chief villager refused to accept to them. Even though they weren't accepted, they decided to stay in the village anyway, but when the materials were distributed to build houses, they were not allowed to get these materials. These people are still living in temporary housing. This happened to about 4 to5 families. At first, when they returned, they didn't get anything. **Relief Worker, Female, Working in Labutta Township, Irrawaddy Division (May 5, 2009)**

I have seen forms of discrimination in my area. Even the donors give the same amount for everyone; the village leaders divide it differently by dividing the villagers into rich and poor groups and distributing it that way. Usually it is where the poor people are receiving more than the rich. But people that are closer to the authorities and government always get more than everyone else. **Survivor, Male, from Kungyangon Township (May 3, 2009)**

### Land Confiscation

In some cases, efforts to rebuild livelihoods were further threatened by the confiscation of survivors' land, often to benefit military-run reconstruction efforts as well as for the material gain of the authorities:

During distribution time, though, most of the people didn't get houses. The people who received the housing support were the people who gave donations to the chief, his relatives and those people who had good relationships with him. Only these people received the new housing. So until now, most of the villagers from there have to stay with a tarpaulin shelter. But, this type of shelter is too hot for them and when it's raining it's not safe for them. When they built the new houses, though, the village leader decided where the new houses would be built and a lot of the time it was built on top of the villagers' garden, the main garden area that they used for their livelihood. There was no discussion about this, but it was just done by the authorities. Also, they were not given any financial compensation for their land being taken. I don't know how much land exactly, how many Rai [1 Rai = 1600 sq. meters], but I can say that it was a lot. This happened to most of the villagers there, that their land was taken for new buildings for other people - the people close to the authorities. None of the villagers had any opportunity to speak out against this. **Relief Worker, Male, Working in Labutta Township, Irrawaddy Division (May 7, 2009)**

## Discussion and Conclusions

In 2006, in response to a lack of focus on human rights protections following natural disasters, the UN Human Rights Council issued the *Report of the Representative of the Secretary-General on human rights of internally displaced persons *[46]. The document recognized the importance of human rights considerations in the context of natural disasters and the humanitarian response:

5. Human rights are the legal underpinning of all humanitarian work pertaining to natural disasters. There is no other legal framework to guide such activities, especially in areas where there is no armed conflict. If humanitarian assistance is not based on a human rights framework, it risks having too narrow a focus and cannot integrate all the basic needs of the victims into a holistic planning process. There is also the risk that factors important for recovery and reconstruction later on will be overlooked. Further, neglecting the human rights of those affected by natural disasters effectively means no account will be taken of the fact that such people do not live in a legal vacuum. They live in countries with laws, rules and institutions that should protect their rights.

The report and its addendum, the *Operational Guidelines on Human Rights and Natural Disasters*, also underscored that protection of human rights and provision of assistance to the populations are the obligation of the government of the affected country. Additionally, the inclusion and consultation with individuals from the affected community during the decision-making process and implementation of relief activities are also fundamental principles set forth in this document. The *Operational Guidelines *further noted the essential role of community members in ensuring "effective, equitable, and sustainable" relief and recovery programs [46,47]. The important role of local communities in disaster responses was also recognized by ASEAN, of which Burma is a member, that called for the promotion of "public participation in programmes related to disaster risk reduction and emergency responses in order to promote community resilience to disasters" and "partnership with relevant stakeholders, including local communities, non-governmental organisations and private enterprises, and strengthen cooperation with United Nationals and relevant international organisations"[48]. In the case of Burma, as reported here, community members were among the first and most effective responders during the crisis.

Unfortunately, as is reported here, there is consensus that donor aid has been and continues to be inadequate for the needs of the affected populations [36,49,50]. Many needs assessments and reports done in partnership with the Burmese government, including reports focused on the social impacts of the storm, have generally not reported on human rights issues, despite the central role that this has been recognized to play[48,51]. The assessment and reporting of human rights concerns is further limited by censorship, and the SPDC tightly controls independent collection and dissemination of information, including reporting on the needs of communities[52,53]. In this context, private and independent relief organizations such as EAT are in unique positions to conduct rights investigations and play an essential role to help ensure "effective, equitable, and sustainable" relief and recovery programs[47]. As members of the communities, they have physical access to the most challenging, heavily-affected areas, especially those where foreign aid access is restricted by the authorities. And, as members of storm-affected communities, they aroused less suspicion while traveling, were trusted by the survivors and volunteer relief workers, and were instrumental in gauging the priorities and needs of the local people. Using participatory methods and operating without the knowledge and consent of the Burmese junta or its affiliated institutions, they were thus well-positioned to serve as independent, community-level monitors of human rights.

These assessments reveal that ongoing human rights abuses have occurred in cyclone-affected areas in the context of relief and reconstruction efforts. These included official interference in relief efforts, harassment and intimidation of private relief workers, misappropriation and confiscation of aid, controls on information, confiscation of land from survivors, and forced labor. Our findings show that the difference of having unhindered access to affected communities and ensuring the anonymity and confidentiality of those willing to testify uncovers realities on the ground that stand in contrast to evaluations conducted in partnership with the ruling junta[27,48,51,54,55].

This assessment was subject to several limitations. Due to security and logistical concerns, it was not possible to conduct a quantitative population-based assessment in tandem with the qualitative human rights investigation. As a result, estimations of the prevalence of human rights abuses were not possible. In addition, the disclosure of identifiers, such as specific village names where the data were collected, was also impossible due to security considerations. To date, at least 21 individuals involved in private cyclone-related activities have been arrested, none associated with this assessment, and some have been sentenced to long prison terms, underscoring the reality of these security concerns in Burma[22]. At least eight additional community activists and cyclone relief volunteers were sentenced to long prison terms in January 2010[56]. Despite such limitations, the use of in-depth interviews, however, offer several strengths to the investigation. Foremost, interviews allow for an understating of the nuanced experiences among survivors and relief workers. The use of qualitative methods also allowed for refinement, addition of questions, and adjustment of the interview guide when new themes emerged over time. Additionally, the ability of the research team to use probes to collect further details of experiences allowed for the verification of veracity and internal consistency, as well as the separation of personal experience from hearsay. An additional limitation was the selected nature of the interviewers and interviewees, and the potential biases inherent in non-random sampling of participants. While population-based approaches would arguably have generated more generalizable findings, this was not feasible for logistical and security reasons; however, non-random approaches, such as used in this investigation, should not undermine the veracity of any individual experience of abuse.

The limitations of this investigation should not detract from the reality that independent community voices in cyclone relief and reconstruction activities should be encouraged. This is particularly important in countries such as Burma, one of the most corrupt governments in the world, and where widespread, systematic human rights abuses of civilians has been widely documented, including violations of several UN Conventions to which Burma is signatory, such as the Convention on the Rights of the Child (CRC), the Convention on the Eradication of All Forms of Discrimination Against Women (CEDAW), and the International Labour Organisation Convention 29 on Forced Labour[17,57-59]. And, even before Cyclone Nargis, strict limitations on international humanitarian assistance, particularly information gathering and travel for international staff, were a reality for aid organizations[60,61]. Community-led monitoring of human rights violations are an important part of assessing responses to complex humanitarian emergencies, particularly where states are unwilling to or have failed to do so.

## Abbreviations used

(ASEAN): Association of South East Asian Nations; (CBO): Community-based Organization; (CHE): Complex Humanitarian Emergency; (EAT): Emergency Assistance Teams-Burma; (ERAT): UN Emergency Rapid Assessment Team; (PONJA): Post-Nargis Joint Assessment; (SPDC): State Peace and Development Council; (TCG): Tripartite Core Group; (USDA): Union Solidarity and Development Association; (UN-HABITAT): UN Human Settlements Programme

## Competing interests

The authors declare that they have no competing interests.

## Authors' contributions

VS contributed to the conception and design, acquisition of data, and interpretation of data; was involved in drafting the manuscript and revising it critically for important intellectual content. NM contributed to the acquisition of data and interpretation of data and was involved in drafting the manuscript. CL contributed to the conception and design, acquisition of data. JL contributed to the conception and design, acquisition of data. AW contributed to the conception and design and interpretation of data, was involved in drafting the manuscript and revising it critically for important intellectual content. BD contributed to the acquisition of data. MM contributed to the conception and design, acquisition of data, and has given final approval of the version to be published. CM contributed to the conception and design, acquisition of data. CB contributed to the conception and design, acquisition of data, and interpretation of data, was involved in drafting the manuscript, and revising it critically for important intellectual content. All authors have read and approved the final manuscript.

## Authors' information

The Emergency Assistance Team (EAT) was established on May 6, 2008, through the collaboration of several Burmese community-based organizations on the Thai-Burma border with years of experience working to provide health and education services, training in human rights, health, education, women's rights, leadership skills and responding to emergencies. EAT works at the grassroots level to provide aid and assistance to the people affected by Cyclone Nargis in the Irrawaddy and Rangoon Division areas.

The Center for Public Health and Human Rights (CPHHR) at the Johns Hopkins Bloomberg School of Public Health uses epidemiologic methods to investigate human rights violations and their impact on the health of individuals, communities and populations. The Center partners with grassroots organizations, human rights groups, and public health researchers and practitioners to address the needs of underserved minorities, ethnic groups facing state violence and discrimination, and stigmatized groups at risk for HIV/AIDS and other epidemic threats.
